# *DLK1* Promotes Lung Cancer Cell Invasion through Upregulation of *MMP9* Expression Depending on Notch Signaling

**DOI:** 10.1371/journal.pone.0091509

**Published:** 2014-03-12

**Authors:** Lin Li, Jinjing Tan, Ying Zhang, Naijun Han, Xuebing Di, Ting Xiao, Shujun Cheng, Yanning Gao, Yu Liu

**Affiliations:** State Key Laboratory of Molecular Oncology, Department of Etiology and Carcinogenesis, Cancer Institute (Hospital), Peking Union Medical College & Chinese Academy of Medical Sciences, Beijing, P. R. China; Harvard Medical School, United States of America

## Abstract

The transmembrane and secreted protein delta-like 1 homolog (*DLK1*) belongs to the EGF-like family. It is widely accepted that *DLK1* plays important roles in regulating cell differentiation, such as adipogenesis and osteogenesis. Aberrant expression of *DLK1* has been found in various types of human cancers, including lung cancer. A previous study in this lab has revealed that *DLK1* is associated with tumor invasion, although the mechanism is still unknown. To explore the potential effects that *DLK1* might have on invasion, *DLK1* was overexpressed or knocked down in the human lung cancer cell lines. The protein's influences on cell invasion were subsequently evaluated. A transwell assay showed that *DLK1* overexpression significantly promoted cancer cell invasion. Western blotting and gelatin zymography analysis indicated that *DLK1* could affect both matrix metalloproteinase-9 (*MMP9*) expression and its extracellular activity. An analysis of *NOTCH1* and *HES1* gene expression and Notch intracellular domain (NICD) nuclear translocation during *DLK1* stimulation or depletion demonstrated that *DLK1* could activate Notch signaling in lung cancer cells. Additionally, the elevated expression of *MMP9* induced by *DLK1* stimulation could be significantly decreased by inhibiting Notch signaling using γ-secretase inhibitor (GSI). The data presented in this study suggest that *DLK1* can promote the invasion of lung cancer cells by upregulating *MMP9* expression, which depends on Notch signaling.

## Introduction

Lung cancer is the leading cause of cancer death worldwide, and tumor metastasis is strongly associated with the prognosis of cancer patients [1], [2]. Our previous studies on differentially expressed genes in human lung squamous cell carcinoma (SCC) with or without lymph node metastasis using DNA microarray analysis found a set of metastasis-associated genes (fdr<0.05, data not shown). Among those genes, *DLK1* showed more than two-fold higher expression in primary tumors with lymph node metastasis, suggesting that *DLK1* may play roles in cancer metastasis. However, both the relationship between *DLK1* and tumor metastasis and its mechanism are poorly characterized.

The delta-like 1 homolog (*DLK1*) gene, with the aliases *FA1*, *ZOG* and *PREF-1*, encodes a transmembrane and secreted protein (DLK1) containing six epidermal growth factor (EGF) domains that is a member of the EGF-like family. This protein is highly homologous to the Notch ligand DLL1 but lacks the Delta/Serrate/Lag (DSL) motif that is critical for interacting with Notch receptors [3], [4]. Studies on *DLK1* have revealed its role in cell differentiation. For example, *DLK1* may function in modulating adipogenesis [5], [6], [7], regulating osteoblast differentiation [8], [9] and inhibiting the differentiation and proliferation of hematopoietic cells [10]. The nonclassical ligand *DLK1* was found to be aberrantly expressed in several human cancers, including neuroblastoma [11], hepatocellular carcinoma [12], [13], gliomas [14] and human prostate cancer [15]. Our previous work also found that *DLK1* is highly expressed in human non-small cell lung cancer and functions as an oncogene [16]. Upregulated expression of *DLK1* in non-small cell lung cancer is associated with lymph node metastasis, but the mechanism is still unknown.

The Notch pathway is a well-known signal transduction pathway during the developmental process and cell fate determination. Although lacking the DSL motif, *DLK1* has been shown to act as an inhibitor of Notch signaling in vitro [17], [18]. Both membrane-bound and secreted DLK1 can interact with NOTCH1 [17], leading to altered cellular distribution of NOTCH1 and inhibition of Notch-regulated gene expression [4], [5], [19]. It has been reported that *NOTCH1* may regulate the expression of matrix metalloproteinase-9 (*MMP9*) in prostate cancer cells, which plays key roles in cancer invasion [20]. Taking these findings together, we hypothesize that Notch signaling might be involved in *DLK1*-induced cancer cell invasion.

The study that we present here provides evidence suggesting that *DLK1* enhanced the ability of lung cancer cells to invade the extracellular matrix (ECM), which validated our previous gene expression profiling results derived from microarray analysis. Furthermore, our data demonstrated that *DLK1* promoted cancer cell invasion through upregulation of *MMP9* expression and enhancement of its extracellular activity, which are dependent on the Notch signaling pathway.

## Materials and Methods

### Cell cultures and treatment

The human lung cancer cell line H520, H1299 and A549 were obtained from the American Type Culture Collection (ATCC, Manassas, VA). The cells were cultivated in RPMI 1640 medium (Life Technologies, Grand Island, NY) with 10% fetal bovine serum (FBS) and 100 μg/ml penicillin-streptomycin in a humidified incubator with 5% CO_2_ at 37°C. The Notch inhibitor L-685,458 (Sigma-Aldrich, St. Louis, MO) was added to the culture media 12 hours after transient transfection with the *DLK1* expression plasmid or the null vector (pcDNA3.1), with a final effective concentration of 5 μM in RPMI 1640 medium containing 1% FBS.

### *DLK1* expression plasmid and small interfering RNA (siRNA)

The *DLK1* eukaryotic expression plasmid was constructed and then stably transfected into H520 cells, as previously described [16]. The *DLK1* expression plasmid was also transiently transfected into H520 and H1299 cells using Lipofectamine-2000 transfection reagent (Invitrogen, Carlsbad, CA); and an Invitrogen Stealth siRNA duplex (Carlsbad, CA) against *DLK1* was utilized for gene silencing using Lipofectamine RNAiMAX reagent (Invitrogen, Carlsbad, CA), following the manufacturers' instructions.

### In vitro ECM invasion assay

Cells either stably (H520) or transiently (H1299) transfected with *DLK1* or null vector were cultured separately until 80% confluence. The cells were then washed three times with phosphate-buffered saline (PBS) and cultured in serum-free media overnight before being subjected to an ECM invasion assay in vitro. The chemoinvasion assay was conducted using BioCoat Matrigel Invasion Chambers with 8 μm pores (BD Biosciences, Bedford, MA) according to the manufacturer's instructions. Briefly, 2.5×10^4^ cells were resuspended in fresh serum-free media and seeded into the upper chamber of a 24-well transwell plate, while the lower chamber contained fresh culture media with 20% FBS as a chemoattractant. The cells were allowed to invade for 22 hours (37°C, 5% CO_2_ atmosphere), and the chambers were then washed with PBS. Those cells that did not invade through the membrane were removed. The invading cells on the lower surface of the membrane were fixed with cold methanol, stained with 0.2% crystal violet and examined. The cells on each membrane were counted in no less than five fields under a light microscope.

### RNA extraction and quantitative reverse transcription-PCR

Total RNA was isolated from the cells with TRIzol reagent (Invitrogen, Carlsbad, CA), and 1 μg total RNA was used for reverse transcription with a SuperScript II reverse transcription kit (Invitrogen, Carlsbad, CA) according to the manufacturer's instructions. Real-time PCR analysis of *MMP9* and *HES1* expression was conducted with the following primers: MMP9-F 5′-TTTGACAGCGACAAGAAGTG-3′, MMP9-R 5′-CAGGGCGAGGACCATAGAGG-3′, HES1-F 5′-TAGCTCGCGGCATTCCAAG-3′ and HES1-R 5′-AAGCGGGTCACCTCGTTCA-3′. The housekeeping gene *18S* ribosomal RNA was picked as an internal control in this study, with the primers as 18S-F 5′-GAAACGGCTACCACATCC-3′ and 18S-R 5′-ACCAGACT2TGCCCTCCA-3′. A SYBR Premix Ex Taq kit (TaKaRa, Shiga, Japan) was used for real-time PCR analysis with a standard amplification protocol: 95°C for 10 s, followed by 40 cycles of 95°C for 5 s and 60°C for 30 s and a final extension at 72°C for 3 min. After the amplification, a standard melting curve procedure was performed for each gene to examine the specificity of amplification.

### Western blotting analysis

Total protein was extracted from approximately 2×10^6^ cultured cells with RIPA buffer, and nuclear protein was extracted using NE-PER nuclear and cytoplasmic extraction reagents (Pierce, Rockford, IL) following the manufacturer's supplied protocol. In total, 40–80 μg of the proteins was separated by electrophoresis and then transferred onto a PVDF membrane, as previously described [21]. The membrane was blocked with 5% defatted milk and incubated with a primary antibody against DLK1 (Proteintech Group, Chicago, IL), NOTCH1 (Cell Signaling Technology, Danvers, MA), cleaved NOTCH1 (Val1744, Cell Signaling Technology, Danvers, MA) or MMP9 (Aviva Systems Biology, San Diego, CA). The anti-Notch intracellular domain (NICD) antibody used in this study can only recognize cleaved and activated NOTCH1 but not full-length NOTCH1. After washing with PBS containing 0.1% Tween-20 (PBST), the membrane was incubated with horseradish peroxidase-conjugated secondary antibody (Dako, Glostrup, Denmark), and the abundance of proteins was detected with chemiluminescence reagents. The membrane was reprobed with an antibody against GAPDH (KangChen Bio-Tech, Shanghai, China) or TBP (Abcam, Cambridge, MA) as internal controls for equivalent protein loading.

### Gelatin zymography

Stably *DLK1*-expressing H520-dlk1 cells, together with null-control H520-pcdb and parental H520 cells, were cultured separately in 10 cm dishes until 80% confluence. The conditioned media were collected and concentrated using an Amicon filter (Millipore, Bedford, MA) according to the manufacturer's protocol. In total, 20 μg of concentrated proteins was electrophoresed under nonreducing conditions, and the gelatinolytic activity of MMP9 was then analyzed using zymography reagents (Bio-Rad, Hercules, CA) following the manufacturer's instructions.

### Statistical analysis

Differences between counts of invading cells in each group in the invasion assay and differences in relative expression in the real-time PCR analysis were evaluated using a Student's t-test. All tests were two-sided, and a p-value<0.05 was considered statistically significant. All statistical tests were performed with the SPSS software package, version 13.0 (SPSS, Chicago, IL).

## Results

### Overexpression of *DLK1* enhanced ECM invasion by lung cancer cells

**Figure 1:**
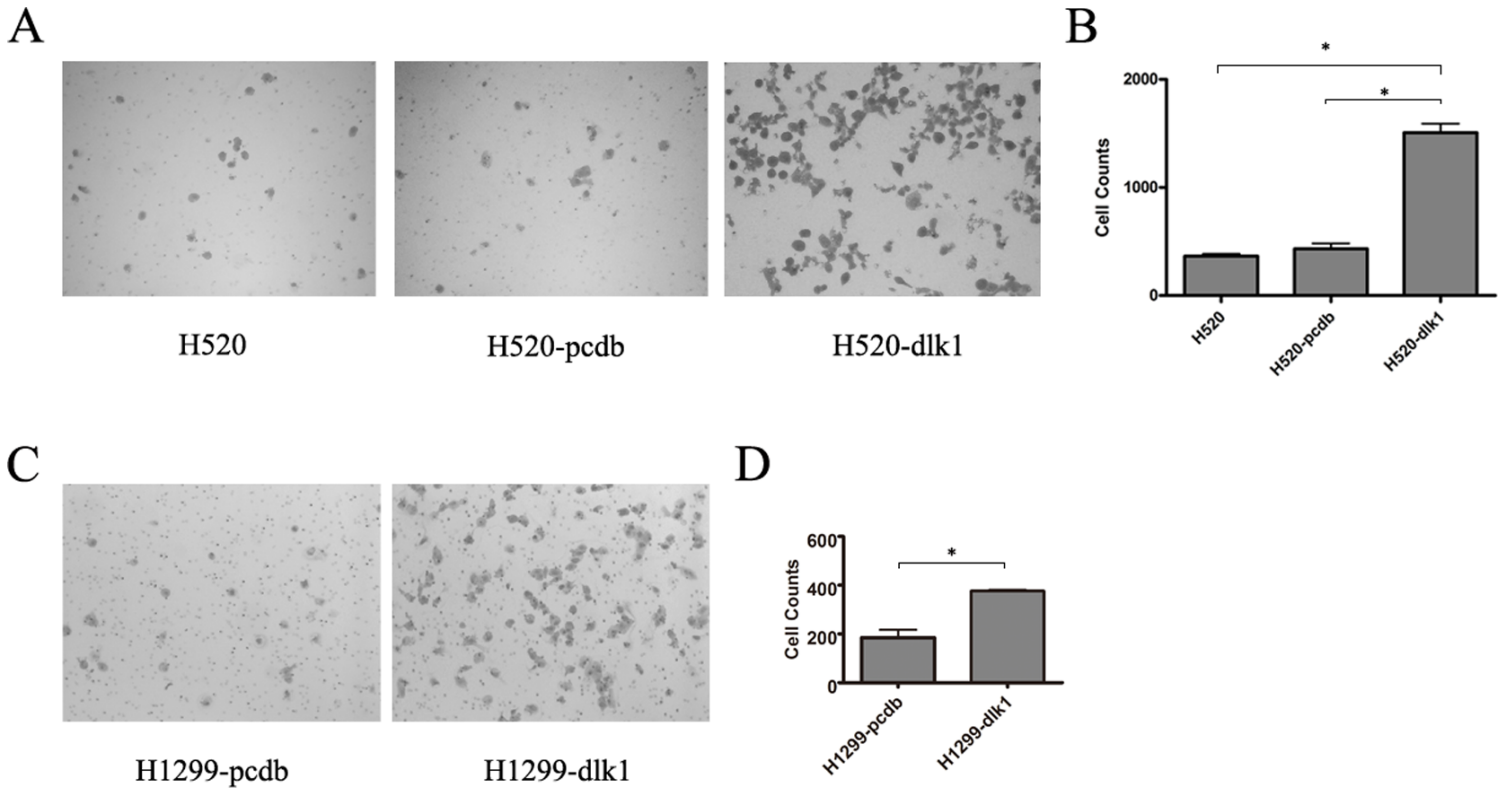
Effect of *DLK1* overexpression on ECM invasion by the human lung cancer cells H520 and H1299 in an in vitro chemoinvasion assay. A, representative photomicrographs of the invading cells that migrated through the Matrigel-coated membrane of the transwell, taken from three groups: H520, the parental cells, H520-pcdb, the null vector-control cells and H520-dlk1, the cells stably expressing DLK1. B, a histogram for the counts of migratory cells from the H520, H520-pcdb and H520-dlk1 groups. C, representative photomicrographs showing H1299 cells, which were transiently transfected with *DLK1* (H1299-dlk1) or null vector (H1299-pcdb), that invaded through the Matrigel-coated membrane of the transwell. D, a histogram for the counts of migratory cells from the H1299-dlk1 and H1299-pcdb groups. The experiments were performed in triplicate (* t-test, p-value <0.05).

To address whether *DLK1* has potential roles in lung cancer metastasis, the lung cancercell lines H520 and H1299 were employed as in vitro model in which endogenous expression of *DLK1* was lacking. H520 cells with stable expression of exogenous *DLK1* (H520-dlk1) were generated previously, together with the H520-pcdb cells, stably transfected with the vector (pcDNA3.1) as the null control [16]. H520-dlk1 cells were subjected to the invasion assay, with H520-pcdb and parental H520 cells as controls. As shown in Figure 1 panel A and B, after a 22-hour period of invasion, there were significantly more H520-dlk1 cells invaded through the chamber membrane coated with Matrigel, compared with H520 and H520-pcdb cells (p-value <0.05). A similar phenomenon was observed in H1299 cells. More *DLK1* transiently transfected H1299 cells (H1299-dlk1) were found invaded through the membrane compared with H1299 cells transfected with the null vector (H1299-pcdb), as shown in Figure 1C and D. These results suggesting that the overexpression of *DLK1* could remarkably enhance cells' ability to invade the ECM.

### Overexpression of *DLK1* upregulated MMP9 expression and activity

**Figure 2:**
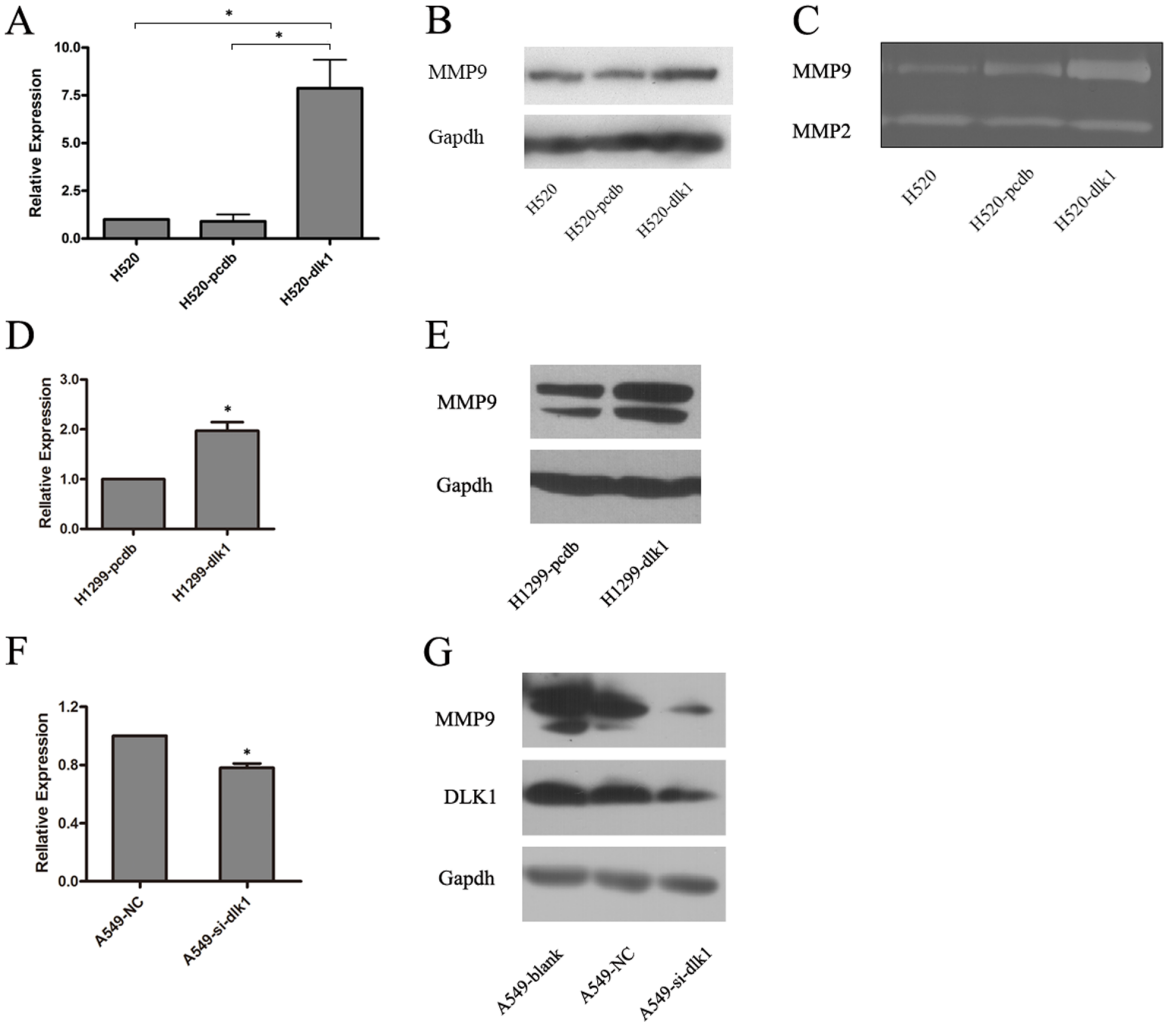
Effect of *DLK1* on the expression and activity of MMP9 in human lung cancer cell lines. A, a histogram for the relative mRNA expression of *MMP9* in H520 cells transfected with *DLK1* (H520-dlk1) or null vector (H520-pcdb) detected by real-time PCR analysis. The expression level of *MMP9* in the blank H520 cells was used as a control and set to 1. B, the protein expression of MMP9 in H520-dlk1, H520-pcdb and H520 cells evaluated by Western blotting analysis. C, gelatin zymography showing the activity of secreted MMP9 and MMP2 in H520-dlk1, H520-pcdb and H520 cells. D, real-time PCR analysis of the relative mRNA expression of *MMP9* in H1299 cells transfected with *DLK1* (H1299-dlk1) or null vector (H1299-pcdb) shown in a histogram. E, Western blotting showing the protein expression of MMP9 in H1299-dlk1 and H1299-pcdb cells. F, a histogram showing the relative mRNA expression of *MMP9* in *DLK1* siRNA (A549-si-dlk1) or null control siRNA (A549-NC) transfected A549 cells evaluated by real-time PCR analysis. G, the protein expression of MMP9 in A549-si-dlk1, A549-NC and blank A549 cells detected by Western blotting. All of the experiments were performed in triplicate. *18S* ribosomal RNA was used as an internal control in the real-time PCR analysis, whereas GAPDH was used as an internal control in the Western blotting analysis (* t-test, p-value <0.05).

Based on the hypothesis that *DLK1*-induced cancer cell invasion was mediated by upregulation of *MMP9*, the expression of *MMP9* upon *DLK1* stimulation was analyzed by both real-time PCR and Western blotting. The results showed that *MMP9* expression was enhanced in H520-dlk1 cells compared with H520 and H520-pcdb cells on both the mRNA and the protein levels (Figure 2A and B). A same trend was also observed when *DLK1* was overexpressed in another lung cancer cell line H1299. After transiently transfected with *DLK1*, highly expressed *MMP9* was detected in both mRNA and protein levels (Figure 2D and E). As MMP9 protein fulfills its function in an activated form in the extracellular space, conditioned media were collected, and gelatin zymography was employed to test MMP9 activity. In Figure 2C, it is shown that *DLK1* could also enhance MMP9 activity in the extracellular space, whereas the activity of MMP2 was unchanged. To further confirm the relationship between *DLK1* and *MMP9*, RNA interference was employed to deplete *DLK1* expression in A549 cells, and *MMP9* expression was examined sequentially. The results showed that DLK1 expression was effectively suppressed by RNAi at the protein level (Figure 2G), and MMP9 expression was significantly inhibited at both the mRNA and the protein levels (Figure 2F and G). These results suggested that *DLK1* might promote cell invasion through regulating MMP9 expression and activity.

### Overexpression of *DLK1* activated Notch signaling pathway

**Figure 3:**
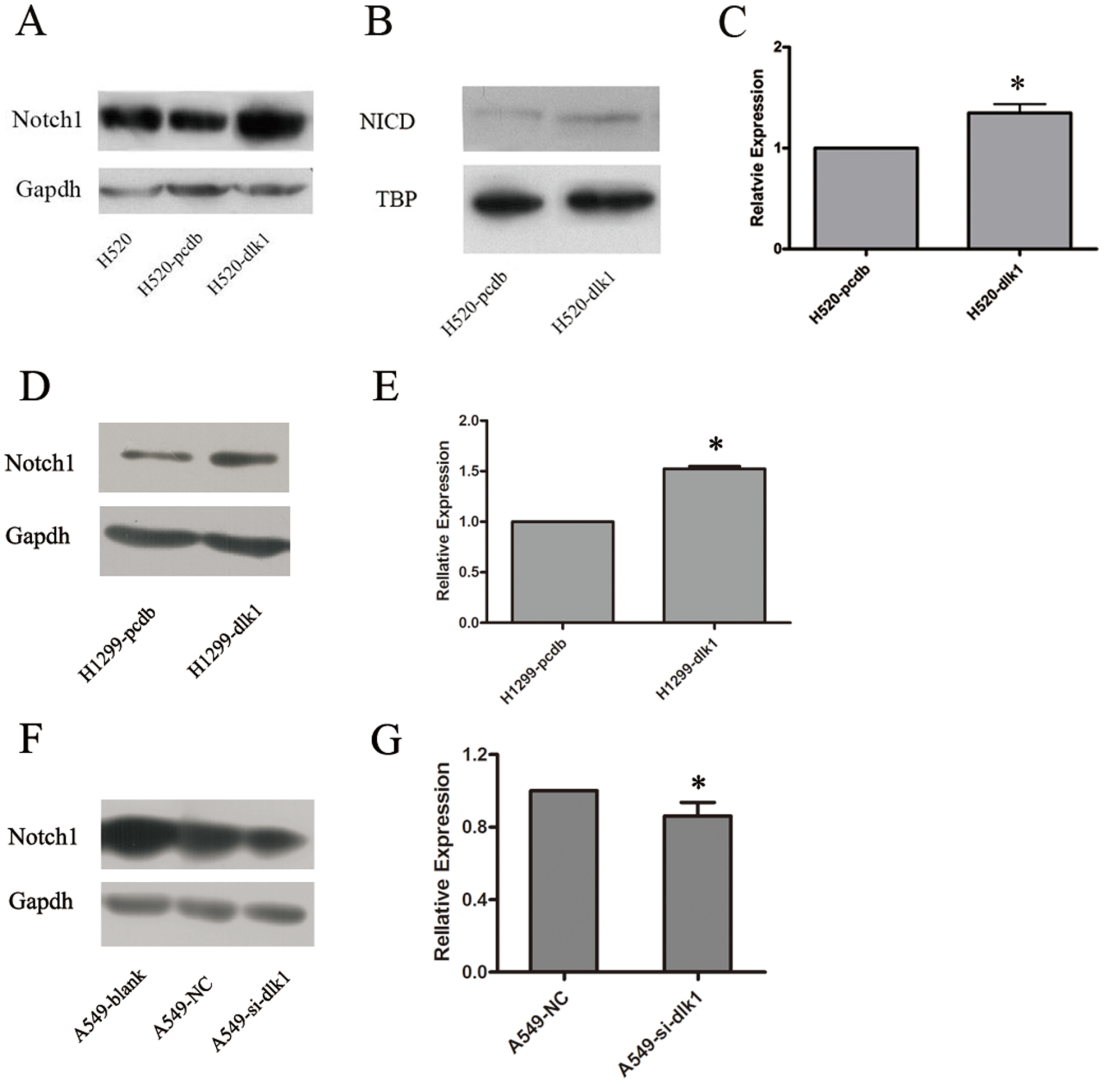
Effects of *DLK1* on the Notch signaling pathway in the human lung cancer cells. A, Western blotting showing NOTCH1 expression in H520 cells after transfection with *DLK1* (H520-dlk1) or null vector (H520-pcdb)examined in whole-cell lysates. GAPDH was used as an internal control. B, Western blotting evaluating nuclear NICD expression in H520-dlk1 andH520-pcdb cells. TBP was used as an internal control. C, a histogram for the relative mRNA expression of Notch target gene *HES1* in the H520-dlk1 and H520-pcdb cells detected by real-time PCR analysis. H520-pcdb cells were used as a control, and its expression level of *HES1* was set to 1. *18S* ribosomal RNA was used as an internal control. D, Western blotting showing the expression of NOTCH1 in H1299 cells transfected with *DLK1* (H1299-dlk1) or null vector (H1299-pcdb). GAPDH was used as an internal control. E, a histogram presenting the relative mRNA expression of *HES1* in H1299-dlk1 and H1299-pcdb cells detected by real-time PCR analysis. F, the expression of NOTCH1 in cytoplasm were evaluated by Western blotting in A549 cells transiently transfected with *DLK1* siRNA (A549-si-dlk1) or null control siRNA (A549-NC). GAPDH was used as an internal control. G, real-time PCR analysis of the relative mRNA expression of *HES1* in A549-si-dlk1 and A549-NC cells, and presented in a histogram. The experiments were performed in triplicate (* t-test, p-value <0.05).

To confirm the association between *DLK1* and *NOTCH1*, we first tested NOTCH1 expression in whole-cell lysates by Western blotting. It was found that overexpression of *DLK1* could upregulate NOTCH1 expression in both H520 and H1299 cells (Figure 3A and D). Because NOTCH1 activation is followed by its cleavage into the NICD and translocation of the NICD into the nucleus for further transcriptional regulation, the amount of NICD in nuclei could reflect Notch pathway activity. We extracted nuclear proteins from H520 cells transiently transfected with *DLK1* or the null-vector and examined the amount of NICD by using Western blotting. The results showed that NICD translocated into nuclei more intensely after overexpression of *DLK1* compared with the null control (Figure 3B). Furthermore, the expression of *HES1*, an explicit Notch pathway target gene, was also examined by real-time PCR in H520 and H1299 cells. There was a significant upregulation of *HES1* after stimulation of *DLK1* expression compared with the null control in both cells (p-value <0.05, Figure 3C and E). In contrast, decreased expression of NOTCH1 and HES1 was detected by Western blotting and real-time PCR, respectively, when DLK1 expression was depleted by RNAi in A549 cells (Figure 3F and G).

### *DLK1* regulated *MMP9* expression partially through Notch signaling pathway

**Figure 4:**
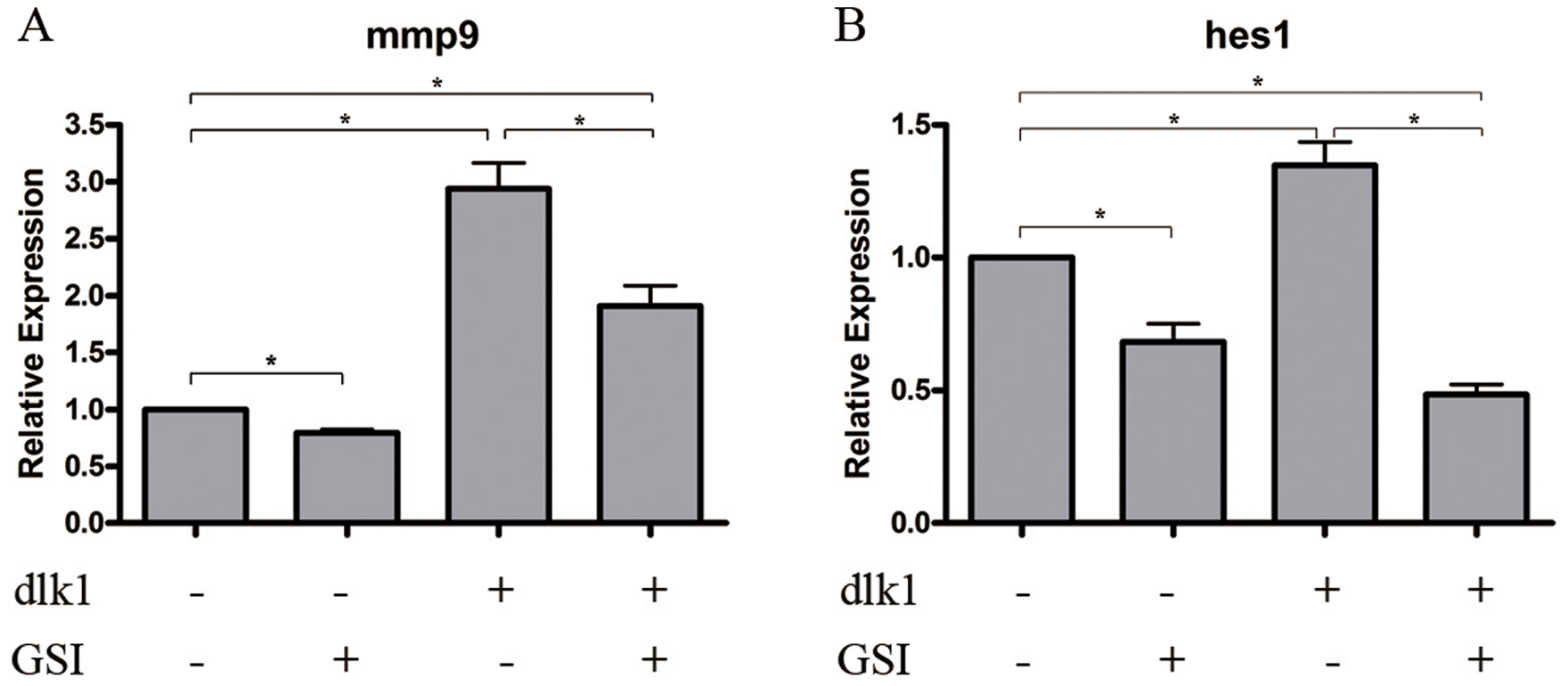
Effects of Notch signaling blockade on *DLK1*-regulated MMP9 expression in the human lung cancer cell line H520. A, a bar graph of the mRNA expression of *MMP9* detected by real-time PCR in H520 cells with or without *DLK1* overexpression and with or without GSI treatment. The group with neither *DLK1* stimulation nor GSI treatment (dlk−/GSI−) was used as a control, and the expression level of *MMP9* was set to 1. *18S* ribosomal RNA was used as an internal control. B, the expression of *HES1* evaluated by real-time PCR in parallel to *MMP9* expression is shown in a bar graph, indicating Notch signaling activities upon *DLK1* overexpression or GSI treatment. The experiments were performed in triplicate (* t-test, p-value <0.05).

To determine whether *DLK1* upregulated *MMP9* expression in a Notch signaling-dependent manner, L-685,458 was applied, which is a γ-secretase inhibitor (GSI) that suppresses NOTCH1 intercellular domain cleavage by γ-secretase, thus inhibiting Notch signaling activity. The relative expression of *MMP9* during stimulation of *DLK*1 expression with or without the GSI treatment was analyzed by real-time PCR. The results showed that when Notch signaling was blocked by treatment with L-685,458, *DLK1*-stimulated *MMP9* upregulation was significantly reduced (Figure 4A), indicating the involvement of Notch signaling in this regulation process. Moreover, it was also noticed that although Notch signaling activity was suppressed to a level comparable with a lack of *DLK1* overexpression (denoted by *HES1* expression in Figure 4B, dlk1+/GSI+ compared with dlk1−/GSI+, t-test p-value  = 0.078), the expression of *MMP9* did not follow the same trend, suggesting that *DLK1* might also regulate *MMP9* expression through signaling transduction pathways other than Notch. The expression of *HES1*, which is a target gene of the Notch signaling pathway, was evaluated in parallel as an indicator of Notch signaling activity (Figure 4B).

## Discussion

Studies on *DLK1* have revealed its functions in cell differentiation and proliferation [5], [6], [10]. It has also been reported that *DLK1* is aberrantly expressed in various types of human cancers, including non-small cell lung cancer [14], [16], [22], [23]. These studies in human cancers mainly focused on the abnormal expression of *DLK1* but did not explore its association with cancer metastasis. Our previous work on metastasis-associated genes in human lung SCC suggested that *DLK1* might function in tumor metastasis (data not shown). In the present study, we validated that overexpression of *DLK1* could enhance the invasive ability of lung cancer cells (as Figure 1 shown), which is consistent with our earlier findings derived from gene expression profiling and extends our knowledge of dlk1's function in human cancer.

Although the DLK1 protein lacks the DSL motif, which is believed to play a key role in ligands' interactions with Notch receptors, an interaction between DLK1 and the NOTCH1 receptor has been shown in a yeast two-hybrid system [19]. However, DLK1's effect on the Notch signaling pathway is still controversial [4], [5], [19]. Baladron et al. suggested that the effect of *DLK1* on Notch signaling is different between *DLK1* variants. Whereas secreted DLK1 may function as a Notch antagonist, membrane DLK1 may activate Notch. Our findings showed that a mixture of membrane and secreted DLK1 might activate Notch signaling in human lung cancer cells. The use of a mixture of *DLK1* variants here was performed in an effort to mimic situations in vivo. In addition to Notch signaling activation, we also observed an upregulation of NOTCH1 expression during stimulation of *DLK1* overexpression. Notably, the activation of Notch signaling by *DLK1* may be a combined effect of the upregulation of NOTCH1 expression and NOTCH1 cleavage stimulated by DLK1 binding. Additionally, aberrant expression of NOTCH1 is found in various types of human cancers [24], [25], including non-small cell lung cancer [26], [27]. Because our study suggests a regulatory relationship between *DLK1* and *NOTCH1*, it is natural to hypothesize that the abnormal expression of NOTCH1 in cancers is partially the consequence of aberrant expression of DLK1.

We found that the *DLK1*-promoted invasion of lung cancer cells was associated with upregulation of MMP9 expression and its extracellular activity (Figure 2), which are involved in ECM breakdown and highly associated with tumor invasion [20]. We further examined whether Notch signaling was involved in *DLK1*-regulated MMP9 expression using a GSI to block Notch signaling and then evaluating the response of MMP9 during stimulation of *DLK1* expression. We observed a significant decrease in the elevated MMP9 expression when Notch signaling was blocked (Figure 4). Interestingly, we also observed that *DLK1* could still moderately upregulate MMP9 expression when the Notch signaling pathway was blocked, implying that *DLK1* may function in cancer invasion in both Notch signaling-dependent and -independent manners. Despite the interaction of DLK1 and NOTCH1, it has also been reported that DLK1 could function in other signaling pathways. For example, DLK1 may interact with IGFBP1/IGF-1 complexes and activate IGF receptor signaling [6] and could also increase the phosphorylation of MEK/ERK and activate MEK/ERK signaling [28], [29]. Our work also suggested that DLK1 could active NF-κB signaling (data not shown). Considering that signaling pathways in a cell have significant cross-talk and that gene transcription is regulated by a combination of different pathways, which we believe to be both logical and dose dependent, it is not surprising that *DLK1* could enhance cell invasion through multiple pathways.

In conclusion, the data presented in this study proved the role of *DLK1* in cancer invasion and explored the molecular mechanism behind this function, making *DLK1* a new candidate gene in cancer metastasis studies.
